# Omega-3 Fatty Acid Supplementation and Coronary Heart Disease Risks: A Meta-Analysis of Randomized Controlled Clinical Trials

**DOI:** 10.3389/fnut.2022.809311

**Published:** 2022-02-03

**Authors:** ShiChun Shen, Chen Gong, KaiQin Jin, Lei Zhou, Yin Xiao, Likun Ma

**Affiliations:** ^1^Department of Cardiology, The First Affiliated Hospital of USTC, Division of Life Sciences and Medicine, University of Science and Technology of China, Hefei, China; ^2^Department of Pediatrics, The First Affiliated Hospital of Anhui Medical University, Hefei, China; ^3^Department of Cardiology, The Second Affiliated Hospital of Anhui Medical University, Hefei, China; ^4^Department of Cardiology, Jinshan Hospital of Fudan University, Shanghai, China; ^5^Second School of Clinical Medicine of Anhui Medical University, Hefei, China

**Keywords:** coronary heart disease, randomized controlled trial, primary prevention, secondary prevention, omega-3 fatty acid supplementation

## Abstract

**Background:**

The clinical benefits of omega-3 fatty acids (FAs) supplementation in preventing and treating coronary heart disease (CHD) remain controversial. Therefore, this study aimed to investigate the clinical benefits of omega-3 FA supplementation, with special attention given to specific subgroups.

**Methods:**

Randomized controlled trials (RCTs) that compared the effects of omega-3 FA supplementation for CHD vs. a control group and including at least 1,000 patients were eligible for the inclusion in this meta-analysis. The relative risk (RR) of all-cause death, major adverse cardiovascular events (MACEs), cardiovascular death, myocardial infarction (MI), stroke, and revascularization were estimated. We analyzed the association between cardiovascular risk and omega-3 FA supplementation in the total subjects. We focused on the cardiovascular risk compared to omega-3 FA in subgroups with different development stages of CHD, omega-3 FA supplementation application dose, diabetes, and sex. PROSPERO Registration Number: CRD42021282459.

**Results:**

This meta-analysis included 14 clinical RCTs, including 1,35,291 subjects. Omega-3 FA supplementation reduced the risk of MACE (RR; 0.95; CI: 0.91–0.99; *p* for heterogeneity 0.27; *I*^2^ = 20%; *p* = 0.03), cardiovascular death (RR; 0.94; CI: 0.89–0.99; *p* for heterogeneity 0.21; *I*^2^ = 25%; *p* = 0.02), and MI (RR; 0.86; CI: 0.79–0.93; *p* for heterogeneity 0.28; *I*^2^ = 19%; *p* < 0.01), but had no significant effect on all-cause death, stroke, and revascularization. In the subgroup analysis, omega-3 FA supplementation decreased the incidence of MACE and cardiovascular death in acute patients with MI, the risk of MI and stroke in patients with CHD, and the risk of MI in patients with high-risk CHD. 0.8–1.2 g omega-3 FA supplementation reduced the risk of MACE, cardiovascular death, and MI. It was revealed that gender and diabetes have no significant association between omega-3 FA supplementation and MACE risk.

**Conclusions:**

Omega-3 FA supplementation had a positive effect in reducing the incidence of MACE, cardiovascular death, MI. Regardless of the stage of CHD, omega-3 FA supplementation can prevent the occurrence of MI. The 0.8–1.2 g omega-3 FA supplementation alleviated CHD risk more effectively than lower or higher doses.

**Systematic Review Registration:**

https://www.crd.york.ac.uk/prospero/, identifier CRD42021282459.

## Introduction

Omega-3 fatty acids (FAs) are polyunsaturated FA commonly found in marine fish and closely linked to cardiovascular health. Omega-3 FA mainly contains α-linolenic acid (ALA), docosahexaenoic acid (DHA), and eicosapentaenoic acid (EPA). In 1972, Bang and Dyerberg compared the dietary difference and serum lipoprotein levels between Inuit and Danes (1). They found that Inuit were not prone to coronary heart disease (CHD), and they ate a lot of seal and whale meat and also blubber. Meanwhile, an observational experiment confirmed that the inclusion of marine omega-3 FA was negatively correlated with the risk of CHD as well (2). Omega-3 FA may reduce the risk of CHD by antiinflammatory effect, improve vasomotor and endothelial cell function, and lower serum lipoprotein levels (3). In 1994, Lungershausen et al. found that blood pressure and plasma triglycerides (TAGs) were significantly decreased in patients with hypertension following omega-3 FA supplementation treatment (4). The specific mechanism by which omega-3 FA supplementation to reduce TAGs remains unclear. At present, it is generally believed that omega-3 FA supplementation can increase mitochondria β-oxidation and thereby reduced endogenous triglyceride TAG synthesis. Concurrently, omega-3 FA supplementation also increases plasma lipoprotein lipase activity to play a protective role in cardiovascular protection (5, 6). In JELIS trial, a lipid intervention trial initiated by Yokoyama et al., daily treatment with 1.8 g EPA in combination with statins proved to be more effective than statins alone in reducing cardiovascular events in patients with high cholesterol (7).

However, further investigation is required to determine whether omega-3 FA supplementation dose and application in primary prevention or secondary prevention are associated with cardiovascular events. A meta-analysis that was performed by Balk et al. also revealed that omega-3 FA supplementation application could be utilized as an effective lifestyle strategy for preventing CHD (8). This protective effect is positively correlated with the applied dose (8). A meta-analysis, including 13 randomized controlled trials (RCTs) with 1,27,477 subjects, indicated that omega-3 FA supplementation could reduce the risk of MI (RR 0.88, 95% CI: 0.83–0.94) and CVD death (RR 0.92, 95%, CI: 0.88–0.97, *I*^2^ = 6%) (9). Abdelhamid et al. performed a meta-analysis that included 79 RCTs with enrolled 1,62,796 subjects to evaluate the role of omega-3 FA supplementation in preventing CHD (10). They demonstrated that based on low-certainty evidence, omega-3 FA supplementation for 12–88 months could reduce CHD risk and death rate (10). Nonetheless, based on medium to high-certainty evidence, they illustrated that omega-3 FA supplementation has insignificant effect on cardiovascular mortality and events (10). REDUCE-IT was a double-blind, multicenter RCT that aimed to determine the effect of icosapent ethyl on cardiovascular events after providing established statin therapy to patients with CHD or diabetes and other risk factors. The results of REDUCE-IT manifested that compared to the control group, icosapent ethyl can significantly reduce the risk of ischemic events by 25%, including cardiovascular death. Additionally, icosapent ethyl can significantly decrease triglyceride levels, and when combined with statin therapy, it appears to be promising than using statins alone. This appears to be promising that omega-3 FA supplementation can prevent cardiovascular risks, but different results were obtained in the other two large clinical RCTs (11). The STRENGTH trial was the latest large-scale double-blind RCT involving 13,078 subjects with high cardiovascular risks (12). The median treatment time with omega-3 FA supplementation and corn oil in the control group was 38.2 months. However, they eventually exhibited no significant difference in cardiovascular events between the two groups (12). The VITAL trial was a large-scale clinical trial for healthy people. This trial attempted to investigate the effects of omega-3 FA supplementation and vitamin D in the primary prevention of cardiovascular events after a 5-year follow-up. Both the results of VITAL and STRENGTH found that omega-3 FA supplementation had no significant effect on the incidence of cardiovascular events; nevertheless, the secondary endpoints of VITAL revealed that omega-3 FA supplementation reduced the risk of MI by 28% (13). A newly published meta-analysis stated no clinical benefit for omega-3 FA supplementation in preventing cardiovascular risks in healthy people and patients with CHD (14). The aforementioned RCTs and meta-analysis results were inconsistent, posing a challenge to the clinical application of omega-3 FA supplementation to prevent cardiovascular events. It is hypothesized that omega-3 FA supplementation effectively prevents cardiovascular events in some specific cases. As recommended by the American Heart Association (AHA), in individuals with preexisting CHD, heart failure (HF), and reduced ejection fraction (EF), employing omega-3 FA supplementation for CHD prevention is a reasonable treatment option (15).

Our research included RCTs with a large sample size compared with the previous meta-analyses and divided the population enrolled into subgroups of subjects with high risks of CHD, cases with CHD, and patients with acute MI. This study focused on preventing cardiovascular events following omega-3 FA supplementation application. Meanwhile, we focused on cardiovascular risks of the three subgroups after applying omega-3 FA supplementation and explored the association between omega-3 FA supplementation and cardiovascular events, application dose, sex, and having diabetes or not.

## Methods

This meta-analysis followed Preferred Reporting Items for Systematic Reviews and Meta-Analyses (PRISMA) guidelines (16). This meta-analysis was performed using the PRISMA checklist (Supplementary Table S1). The protocol of this meta-analysis was registered on the PROSPERO database (https://www.crd.york.ac.uk/prospero/) with the Registration Number CRD42021282459.

### Search Strategy

The search strategy was conducted in accordance with the participants, intervention, comparison, outcome, and study design (PICOS) format as follows: *P* = adults (above 18 years old) with high risks of CHD or confirmed CHD or MI; *I* = omega-3 FA supplementation; *C* = control group with or without placebos; *O* = all-cause death and cardiovascular outcomes including major adverse cardiovascular events (MACEs), cardiovascular death, myocardial infarction (MI), stroke, and revascularization; *S* = RCT.

Chen Gong and ShiChun Shen independently searched databases including PubMed, Google Scholar, Cochrane library, and Clinicaltrial.gov to screen all the eligible RCTs published before October 01, 2021 without language restrictions. The combination terms of keywords omega-3 fatty acid (FA) supplementation, fish oil, eicosapentaenoic acid (EPA), docosahexaenoic acid (DHA), RCT, CHD, cardiovascular disease, myocardial infarction (MI), sudden cardiac death, and stroke were searched in the above database.

### Inclusion Criteria

- RCTs enrolled adult subjects over 18 years old with high risks of CHD or confirmed CHD or not.
- The sample size of RCTs was more than 1,000.
- RCTs were designed to compare omega-3 FA supplementation to control group with or without placebo.
- Outcomes of RCTs include one of the following events: all-cause death, MACEs, cardiovascular death (CV death), MI, stroke, and revascularization.

### Data Extraction

In each RCT, we extracted trial registration number if applicable, first author, publication year, trial location, participant characteristics, type and dose of omega-3 FA supplementation, treatment duration, subject number of omega-3 FA supplementation group and control group, reported endpoints, and study design. Two researchers independently completed data collection, and if any discrepancies were encountered, they were resolved through negotiation.

Major adverse cardiovascular event was defined as a composite of cardiovascular death, MI, and stroke. MI and stroke were defined as non-fatal MI and non-fatal ischemic stroke, respectively. However, not all RCTs reported the specific definition of MI and stroke. If RCT did not specify the definition of MI and stroke, respectively, as non-fatal MI and ischemic stroke, the number of total MI and stroke reported in that RCT was collected in our meta-analysis. When the results of the same RCT were updated, we decided to report the latest report.

### Assessment of Methodological Quality

We assessed the risk of bias based on Cochrane collaboration tool for the methodological quality of included RCTs (17). Assessment elements of Cochrane collaboration tool include random sequence generation, allocation concealment, participant and personnel blinding, blinding of outcome assessment, incomplete outcome data, no selective outcome reporting and other sources of bias.

### Subgroup Analysis

To further analyze the cardiovascular risks of omega-3 FA supplementation on specific populations, we divided the included experimental populations into subgroups with high risks of CHD, diagnosed with CHD, and with acute MI based on the participants' prior medical history to conduct relevant subgroup analysis. The subgroup of MI included RCTs enrolled subjects with acute MI within 3 months. CHD subgroup included RCTs that enrolled patients with old MI for more than 3 months and confirmed CHD.

A previous study performed by Bernasconi et al. found that cardiovascular mortality and fatal MI prevention can be achieved with < 0.8–1.2 g/d omega-3 FA, and the protective effect quickly plateaued with the increasing dosages (18). According to the treatment doses of omega-3 FA supplementation, we divided the included studies into low-dose studies with dose of < 0.8 g, moderate-dose studies with dose equal to or >0.8 and <1.2 g, and also high-dose studies with dose equal to or more than 1.2 g. The subgroup analyses of diabetes and sex were performed to determine the influencing factors of the cardiovascular effect of omega-3 FA supplementation.

### Statistical Analysis

We used the Peter's test and regression test for funnel plot asymmetry to assess the risk of bias. *I*^2^ was used for the heterogeneity between each RCTs. If *I*^2^ was <50% or *p* for heterogeneity more than 0.10, the fixed-effects model was used, and if *I*^2^ was >50% or p for heterogeneity < 0.10, the random-effects model was used. A sensitivity analysis was conducted to reduce and exclude sources of heterogeneity as follows. (1) We compared the calculation results of the random-effects model and the fixed-effects model to verify the robustness of the results of our research. (2) We eliminated each study in turn to observe the change in *I*^2^. If the value of *I*^2^ was significantly reduced after an RCT was eliminated, then the RCT is the source of heterogeneity. (3) We performed subgroup analysis according to the medical history of CHD, the doses of omega-3 FA supplementation, diabetes or not, and sex. In this meta-analysis, *p* < 0.05 was considered significant. *R* (version 4.1.1) was used to compute statistical tests [relative risks (RRs), confidence intervals, sensitivity analyses, and *I*^2^-test]. Tables and forest plots produced by R (version 4.1.1) were used to show data.

## Results

**Figure 1:**
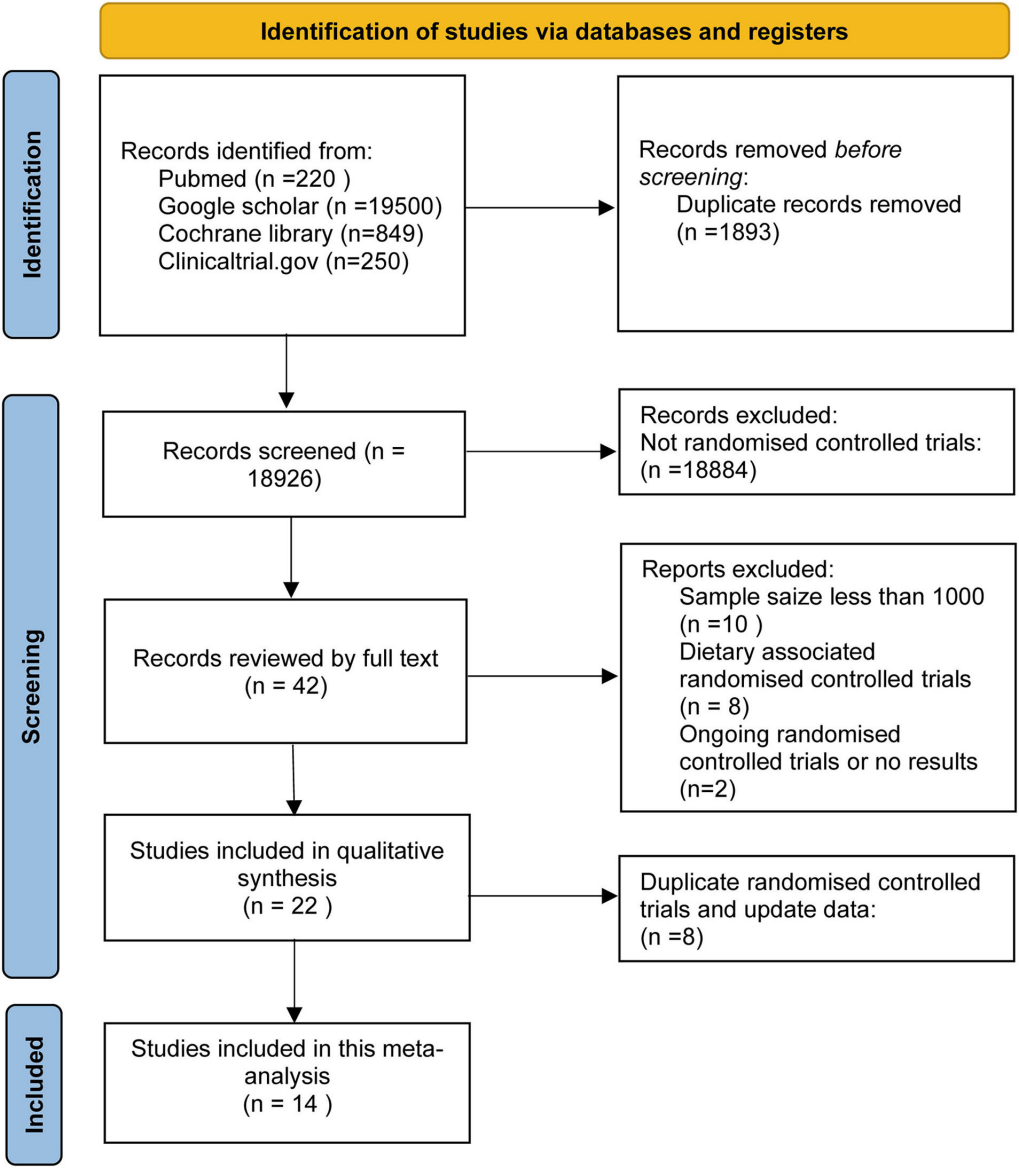
Flow diagram of the study selection process.

This research retrieved 20,819 articles, of which 20,777 were excluded based on the title and abstract. Subsequently, we excluded RCTs for samples <1,000 and assess non-supplement omega-3 FA (e.g., food fortified with omega-3 or dietary advice). Finally, 14 RCTs, including 1,35,291 subjects, were included in this meta-analysis. These subjects include people at high CHD risks, diagnosed with CHD, and patients with acute MI. A total of 67,704 subjects received omega-3 FA supplementation at doses ranging from 0.4 to 4 g, whereas 67,587 cases received control treatment. Figure 1 displays the determination of relevant RCTs and finally retrieved the process of obtaining the final literature. Table 1 shows the characteristics of the finally included 14 RCTs.

**Table 1:** Main characteristics of included RCTs.

**References**	**Trial registration**	**Location**	**Participants**	**Type and dose of omega-3 FA, mg/d**	**Treatment duration, y**	**No. omega-3 FA/control**	**Endpoints assessed**	**Study design**
Marchioli et al. (19)	-	Italy	MI within 3 months	EPA/DHA: 0.85–0.88	3.5	2,835/2,828	All-cause death, non-fatal MI, non-fatal stroke; cardiovascular death	Multicenter, open-label RCTs
Yokoyama et al. (7)	NCT00231738	Japan	With or without CVD	EPA 1.80	4.6	9,326/9,319	Cardiac death, MI	Single-center, open-label RCTs
Tavazzi et al. (20)	NCT00336336	Italy	HF	EPA/DHA 0.85	3.9	3,494/3,481	Cardiovascular death, MI, and stroke	Multicenter, Quadruple-blind RCTs
Rauch et al. (21)	NCT00251134	German	MI with in 3–14 days	EPA/DHA 0.85	1	1,925/1,893	Cardiac death, all-cause death, revascularization	Multicenter, Quadruple-blind RCTs
Kromhout et al. (22)	NCT00127452	Netherlands	Old MI	EPA/DHA 0.40	3.4	2,404/2,433	Non-fatal MI, Non-fatal stroke	Multicenter, Quadruple-blind RCTs
Galan et al. (23)	ISRCTN41926726	France	MI, unstable angina, stroke	EPA/DHA 0.60	4.2	1,253/1,248	Non-fatal MI, stroke, cardiovascular death	Multicenter, double-blind RCTs
Bosch et al. (24)	NCT00069784	Worldwide	Diabetes, MI, stroke, or revascularization	EPA/DHA 0.84	6.2	6,281/6,255	Cardiovascular death, non-fatal MI, non-fatal stroke; all-cause death	Multicenter, open-label RCTs
Roncaglioni et al. (25)	NCT00317707	Italy	Multiple cardiovascular risk factors but not MI	EPA/DHA 0.86	5	6,239/6,266	Death, non-fatal MI, and non-fatal stroke, cardiovascular death	Single center, Quadruple-blind RCTs
Bonds et al. (26)	NCT00345176	USA	Stable, existing CVD	EPA/DHA 1.00	4.8	2,147/2,056	CVD death, MI/stroke/CVD death, revascularization	Multicenter, double-blind RCTs
Bowman et al. (27)	NCT00135226	UK	Diabetes mellitus but not CVD	EPA/DHA 0.84	7.4	7,740/7,740	Non-fatal MI or stroke, revascularization	Single center, Quadruple-blind RCTs
Manson et al. (13)	NCT01169259	USA	Without cancer, stroke, revascularization	EPA/DHA 0.84	5.3	12,933/12,938	MI, stroke, and death from cardiovascular causes	Single center, Triple-blind RCTs
Bhatt et al. (11)	NCT01492361	Worldwide	CVD or with diabetes and other risk factors	EPA 3.60	4.9	4,089/4,090	Cardiovascular death, MI, stroke, coronary revascularization	Multicenter, Triple-blind RCTs
Nicholls et al. (12)	NCT02104817	Worldwide	Adult patients at high risk for CVD	EPA/DHA 3.00	3.2	6,539/6,539	Cardiovascular death, non-fatal MI, non-fatal stroke, revascularization	Multicenter, Triple-blind RCTs
Kalstad et al. (28)	NCT01841944	Norway	MI within 2–3 weeks	EPA/DHA 1.60	2	505/509	All-cause death, non-fatal MI, stroke, revascularization	Multicenter, Triple-blind RCTs

*FA, fatty acid; EPA, eicosapentaenoic acid; DHA, docosahexaenoic acid; MI, myocardial infarction; RCT, randomized controlled trials; CVD, cardiovascular disease; HF, heart failure*.

**Figure 2:**
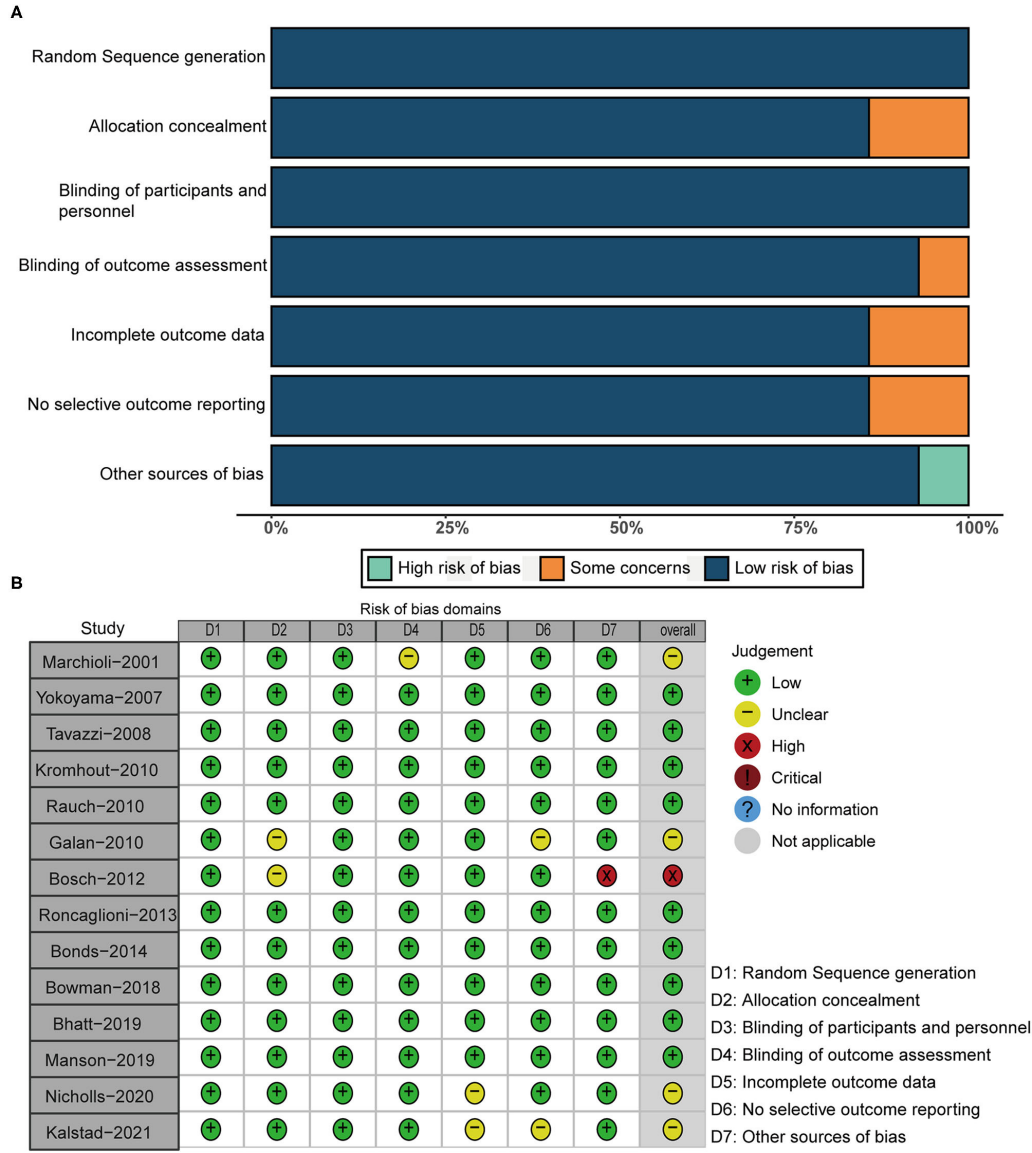
Risk of bias plot. **(A)** Risk of bias summary; **(B)** risks of bias of each included study.

According to the design of each RCT, we used the Cochrane tool to score 14 RCTs for risk of bias. Figure 2 demonstrats the methodological quality for each RCT and showed the risk of bias of RCTs included in our meta-analysis was low.

### Endpoints

**Figure 3:**
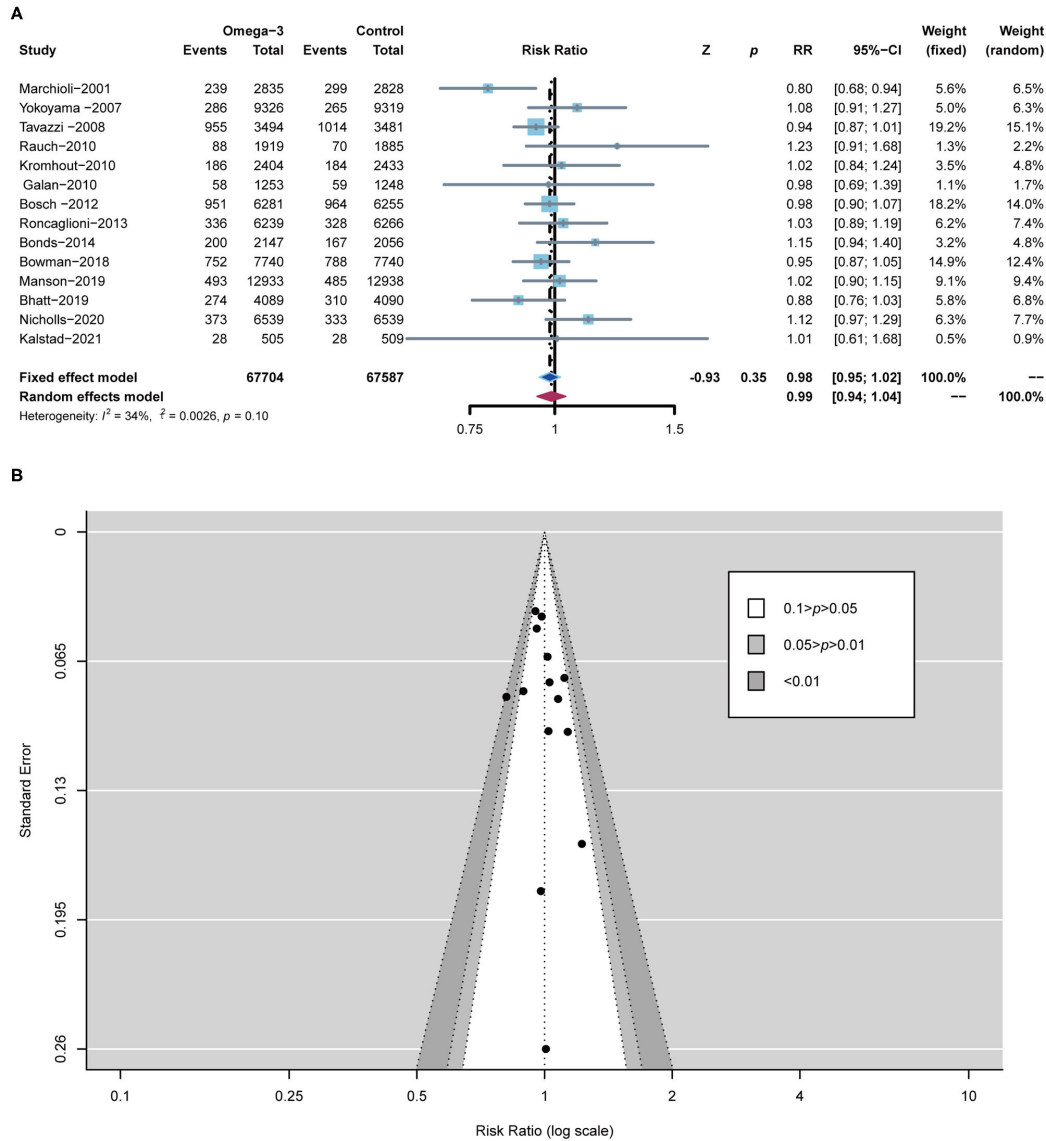
Forest plot of all-cause death and the detection of publication bias. **(A)** Forest plot of all-cause death; **(B)** funnel chart.

All RCTs, including 1,35,291 subjects, reported the occurrence of all-cause death. All-cause death for omega-3 FA-supplemented group (7.71%) was similar to that of the control group (7.83%) (RR 0.98; 95% CI: 0.95–1.02; *p* for heterogeneity 0.10; *I*^2^ = 34%; *p* = 0.35) (Figure 3A). Peter's test and the funnel plot were employed to detect the risk of bias with *p* < 0.05. From the perspective of figure geometry, the funnel chart is symmetrical (Figure 3B). The aforementioned evidence showed that the risk of bias of RCTs included in our meta-analysis was low.

**Figure 4:**
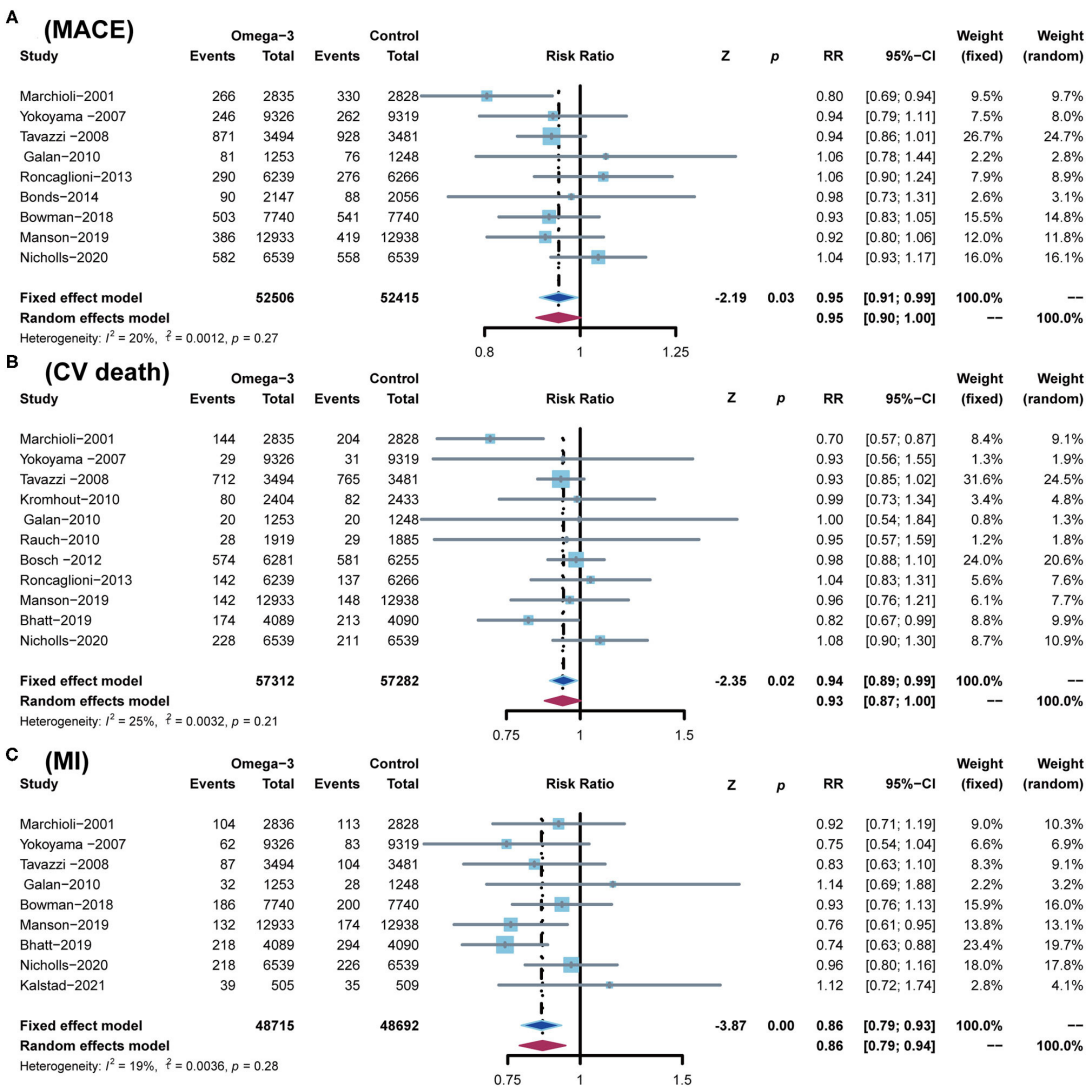
Comparison of omega-3 FA supplementation vs. control group on the risks of **(A)** MACE; **(B)** CV death; **(C)** MI. MACEs, major adverse cardiovascular events; CV death, cardiovascular death; MI, myocardial infarction.

A total of 9 RCTs reported MACE, which was defined as a composite of cardiovascular death (CV death), non-fatal ischemic stroke, and non-fatal MI. A total of 11 RCTs reported CV death, 9 RCTs reported MI, 10 RCTs reported stroke, and 9 RCTs reported revascularization, respectively. In comparison with the control group, omega-3 FA supplementation treatment reduced the risk of MACE by 5% (RR; 0.95; CI: 0.91–0.99; *p* for heterogeneity 0.27; *I*^2^ = 20%; *p* = 0.03), the risk of CV death by 6% (RR; 0.94; CI: 0.89–0.99; *p* for heterogeneity 0.21; *I*^2^ = 25%; *p* = 0.02), and the risk of MI by 14% (RR; 0.86; CI: 0.79–0.93; *p* for heterogeneity 0.28; *I*^2^ = 19%; *p* < 0.01) (Figure 4). However, the incidence of stroke and revascularization was not significantly different between omega-3 FA supplementation treatment group and control group (stroke: RR; 0.97; CI: 0.90–1.05; *p* for heterogeneity 0.23; *I*^2^ = 23%; *p* = 0.49 and revascularization: RR; 0.96; CI: 0.92–1.01; *p* for heterogeneity 0.45; *I*^2^ = 0; *p* = 0.13, respectively) (Supplementary Figure S1).

According to the low heterogeneity of included RCTs, a fixed-effects model was utilized to analyze data. Meanwhile, we used a random-effects model to perform the same analysis. The results analyzed by the random-effects model are consistent with those of the fixed-effects model, confirming the robustness of current results (Figures 3, 4; Supplementary Figure S1).

### Subgroup Analysis

#### Development Stage of CHD

Three RCTs enrolled 10,481 patients who had an MI within the previous 3 months (19, 21, 28), four RCTs enrolled 27,036 patients with confirmed CHD (11, 22, 23, 26), and the other seven studies included 97,774 subjects with high CHD risks (7, 12, 13, 20, 24, 25, 27). If an RCT included subjects with different development stages of CHD and the RCT included the largest number of people at a certain development stage of CHD, then the RCT was included in the corresponding subgroup of the certain development stage of CHD. The JELIS and STRENGTH trials included many subjects with high risks of CHD and also a small number of subjects with confirmed CHD (7, 12). However, no specific subgroup outcomes were reported; therefore, we included them in the subgroup of high risks of CHD. As the number of patients with CHD in REDUCE-IT trial was larger than subjects with high risks of CHD, this RCT was classified into CHD subgroup in our meta-analysis (11). Although ALPHA OMEGA included all patients with MI, we still included them in the CHD subgroup for the participants undergone MI more than 3 months (22).

**Figure 5:**
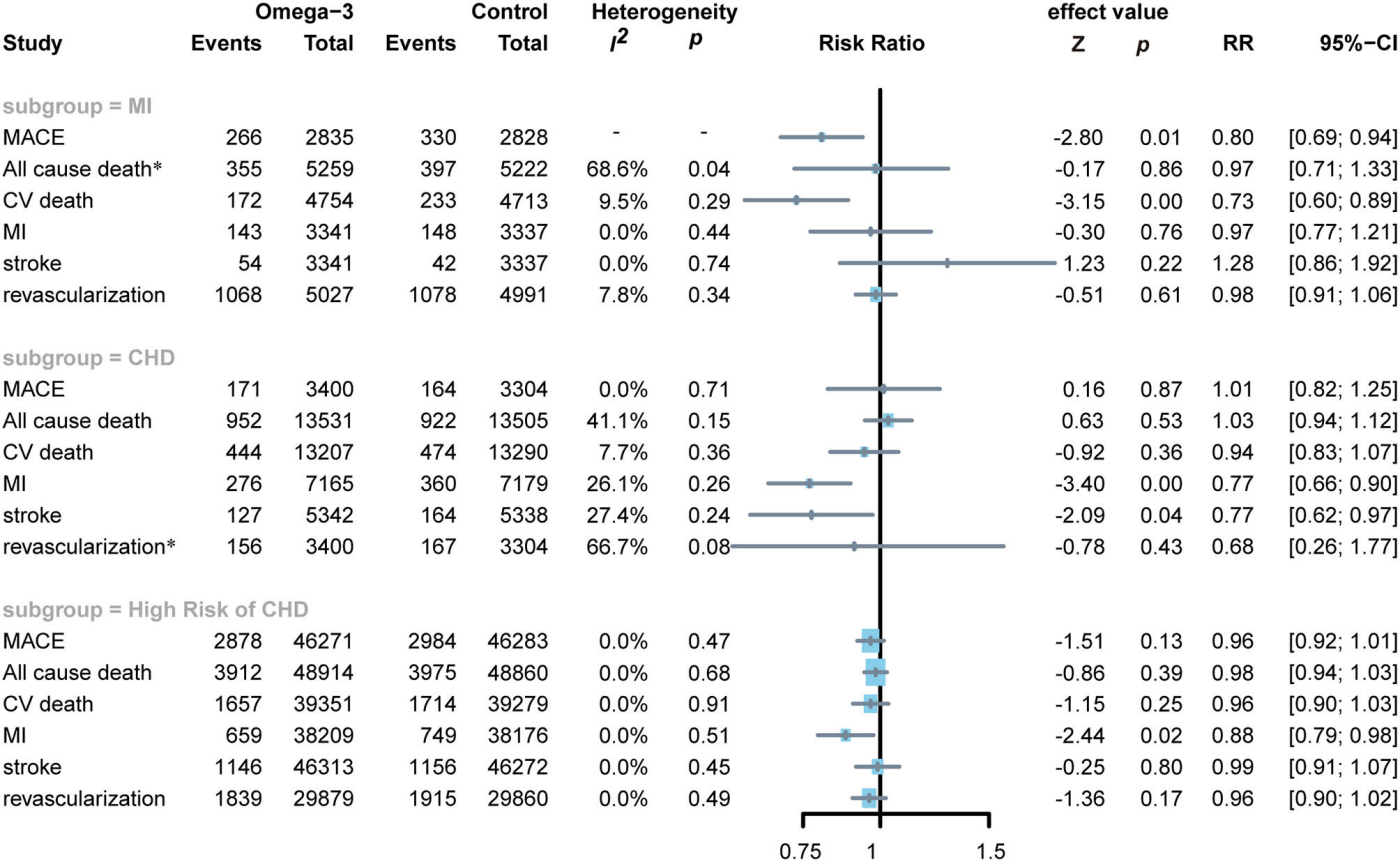
Forest plot of subgroup of subjects with MI, CHD, and high risks of CHD. MI: myocardial infarction; CHD: coronary heart disease; MACEs, major adverse cardiovascular events; CV death, cardiovascular death. *Random-effects model.

In subjects with MI, omega-3 FA supplementation significantly reduced the incidence of MACE (RR0.80; CI: 0.69–0.94; *p* and *I*^2^ for heterogeneity are not applicable for only one RCT reported the MACE; *p* = 0.01) and the risks of CV death (RR 0.73; CI: 0.60–0.89; *p* for heterogeneity 0.29; *I*^2^ = 9.5%; *p* < 0.01), but no significant effect on all-cause death, MI, stroke, and revascularization (Figure 5). Applying omega-3 FA supplementation in patients with CHD can significantly reduce the risks of MI (RR 0.77; CI: 0.66–0.90; *p* for heterogeneity 0.26; *I*^2^ =2 6.1%; *p* < 0.01) and stroke (RR 0.77; CI 0.62–0.97; *p* for heterogeneity 0.24; *I*^2^ = 27.4%; *p* = 0.04), but could not reduce the risks of MACE, all-cause death, cardiovascular death, and revascularization. Besides, omega-3 FA supplementation induced a decrease in MI risks (RR 0.88; CI: 0.79–0.98; *p* for heterogeneity 0.51; *I*^2^ = 0.0%; *p* = 0.02) in subjects at elevated risks of CHD; however, it had no significant effect on the risk of MACE, all-cause death, CV death, stroke, and revascularization.

#### Dose of Omega-3 FA

**Figure 6:**
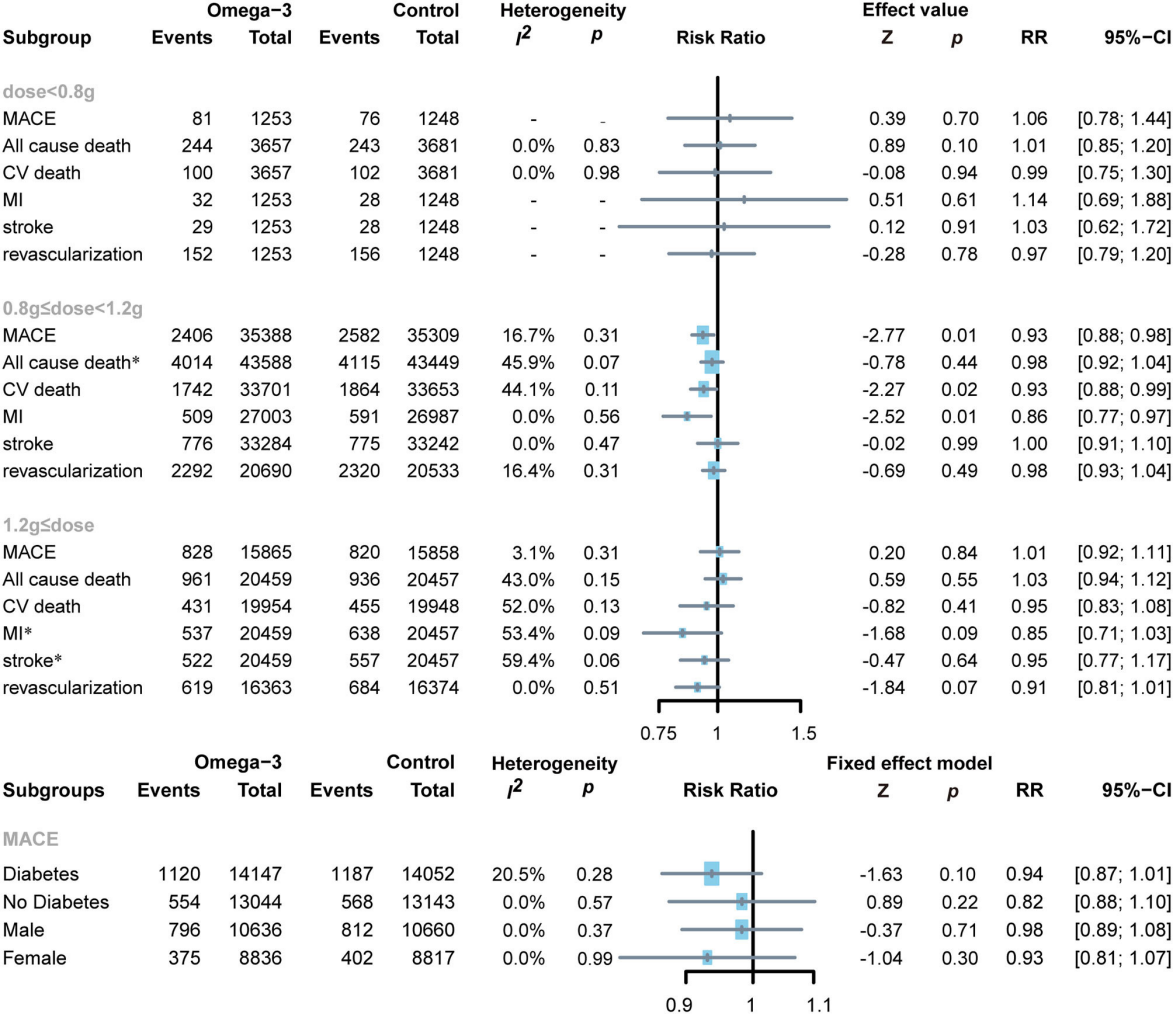
Forest plot of subgroup of the application dose of omega-3 FA, diabetes or not, and sex. MACEs, major adverse cardiovascular events; CV death, cardiovascular death; MI, myocardial infarction. *Random-effects model.

Two RCTs applied low doses of omega-3 FA supplementation on 7,338 subjects (22, 23), the other eight RCTs applied moderate doses of omega-3 FA supplementation on 87,037 subjects (13, 19–21, 24–27), whereas the remaining four RCTs applied high doses of omega-3 FA supplementation on 40,916 subjects (7, 11, 12, 28). To determine the association between omega-3 FA supplementation doses and cardiovascular risk, we conducted a subgroup analysis. Moderate-dose omega-3 FA supplementation treatment reduced the risk of MACE (RR 0.93; 95% CI: 0.88–0.98; *p* for heterogeneity 0.31; *I*^2^ = 16.7%; *p* = 0.01), CV death (RR 0.93; 95% CI: 0.88–0.99; *p* for heterogeneity 0.11; *I*^2^ = 44.1%; *p* = 0.02), and MI (RR 0.86; 95% CI: 0.77–0.97; *p* for heterogeneity 0.56; *I*^2^ = 0.0%; *p* = 0.01) (Figure 6). Treatment with lower and higher dose of omega-3 FA supplementation did not exhibit similar benefits for MACE, MI, and cardiovascular death. Regardless of the dose application of omega-3 FA supplementation, the data of RCTs support that omega-3 FA supplementation has no significant effect on all-cause death, stroke, and revascularization.

#### Diabetes or Not

Two RCTs reported MACE events in patients with diabetes, including 28,199 diabetic subjects and 26,187 non-diabetic subjects (12, 13, 27). Administration of omega-3 FA supplementation in patients with diabetes or non-diabetes was proven to have no significant effect (diabetes: RR 0.94; 95% CI: 0.87–1.01; *p* for heterogeneity 0.28; *I*^2^ = 20.5%; *p* = 0.10 and no diabetes: RR 0.82; 95% CI: 0.88–1.10; *p* for heterogeneity 0.57; *I*^2^ = 0.0%; *p* = 0.22, respectively) (Figure 6).

#### Sex

Two RCTs reported the number of MACE events in male and female subjects (12, 13). A total of 21,296 male subjects and 17,653 female subjects were enrolled. It can be observed from the results that gender has no significant impact on MACE after applying omega-3 FA supplementation (men: RR 0.98; 95% CI: 0.89–1.08; *p* for heterogeneity 0.37; *I*^2^ = 0.0%; *p* = 0.71 and women: RR 0.93; 95% CI: 0.81–1.07; *p* for heterogeneity 0.99; *I*^2^ = 0.0%; *p* = 0.30, respectively (Figure 6).

## Discussion

This research aimed to synthesize existing large-scale clinical trials and analyze the impact of omega-3 FA supplementation on cardiovascular risks. The included 14 RCTs that comprise trials with subjects at different development stages of CHD and used different omega-3 FA supplementation doses as interventions and with or without placebos as controls. It is commendable that all 14 RCTs had good methodological quality.

This meta-analysis demonstrated that additional omega-3 FA supplementation could decrease the incidence of MACE, cardiovascular death, and MI. However, insignificant effect on all-cause death, stroke, and revascularization was observed between omega-3 FA supplementation and the control group. Our subgroup analysis found that omega-3 FA supplementation can reduce the risks of MACE and MI in people with acute MI, the risk of MI and stroke in people with CHD, and the risk of MI in patients with high risks of CHD. In addition, according to the used omega-3 FA dose in RCTs, we found that moderate-dose omega-3 FA ranging from 0.8 to 1.2 g attenuated the incidence of MACE, cardiovascular death, and MI. However, lower and higher dose omega-3 FA supplementation did not show a similar advantage in reducing the risk of MACE, cardiovascular death, and MI. A wide range of studies illustrated that cardiovascular benefits of omega-3 FA supplementation are positively correlated with the given dose (26). There was a controversy between this study results and previously reported findings, which may be associated with the baseline omega-3 index (EPA+DHA in red blood cells) (29). A baseline omega-3 index >8% implied a lower risk of cardiovascular (30). In the high-dose subgroup, OMEMI study that was performed by Kalstad et al. was mainly conducted in Norway, where fish consumption is high. Since the daily diet is rich in omega-3 FA, it is evident that the subjects' baseline omega-3 index is significantly higher. The results of OMEMI experiment revealed that 1.8 g n-3 PUFAs daily for 2 years was used to treat elderly patients with a recent AMI that did not reduce incidence of cardiovascular events or all-cause death. Studies also revealed that a daily dose of < 0.8–1.2 mg omega-3 FA supplementation can reduce the risks of cardiovascular death and MI (18, 29, 31). As the dose increases, it will not increase the cardiovascular benefits but rather may increase the risk of malignant arrhythmias such as atrial fibrillation (AF) (18). In STRENGTH and OMEMI experiments, high omega-3 FA supplementation doses increased the risk of AF. As this adverse impact of excessive doses becomes more prevalent, the cardiovascular advantages of omega-3 FA supplementation may gradually stabilize or even diminish at moderate to high doses (32).

Omega-3 FA is mainly found in marine fish. Previous epidemiological studies stated that compared to non-Mediterranean diets, a Mediterranean diet containing a significant amount of omega-3 FA alleviates cardiovascular risks (33, 34). In people on a non-Mediterranean diet, even a slight amount of fish diet can also reduce cardiovascular risk (35). GISSI-P included Italians within 3 months of MI (19). They were on a Mediterranean diet and received omega-3 FA supplementation to reduce the risk of death from MI. Patients with MI for 3–14 days were enrolled in OMEMI. In addition, an insignificant difference was observed in cardiovascular events after receiving omega-3 FA supplementation or not (28). In that study, 73.2% of subjects ate fish at least once a week during the study period, significantly reducing the incidence of cardiovascular events. Eating fish once per week has been demonstrated to reduce the risk of cardiovascular events by 52% compared to eating fish once per month in a previous prospective study, which enrolled 20,551 subjects (36). In the STRENGTH experiment, all subjects received 4 weeks of statin treatment before grouping, and no significant difference was observed in cardiovascular events between omega-3 FA supplementation treatment and the control group (11).

Aung et al. reported the association between omega-3 FA supplementation and cardiovascular events in patients with a history of MI (37). The RCTs enrolled subjects with the medical history of MI, and some who had acute MI were included in their meta-analysis (37). However, these subjects had MI, and the time of omega-3 FA supplementation use after MI was quite different, which is a significant factor in reporting biased results. Their meta-analysis demonstrated that omega-3 FA supplementation utilization in patients with MI had no influence on all-cause death, cardiovascular death, MI, any cardiovascular events, and stroke compared to applying placebo (37). In our meta-analysis, we divided patients with MI into old and acute stages according to the occurrence time of MI to decrease the bias. Our subgroup analysis found that omega-3 FA supplementation significantly reduced the risk of MACE and MI in patients with acute MI. This is probably because we had a clear definition of the acute MI subgroup, but previous meta-analyses did not subdivide the time of MI. The difference between old MI and acute MI was directly included in their study. In Casula's research (38), omega-3 FA supplementation was found to be alleviated the risk of MI in patients with patients with CHD, which is compatible with our results.

A recent meta-analysis conducted by Rizos et al. performed a prognostic event analysis based on the dose of omega-3 FA supplementation administrated in each included study (13). The meta-analysis reported outcomes including all-cause deaths, cardiovascular death, sudden death, MI, and stroke. In contrast to our research results, they disclosed that the application dose of omega-3 FA supplementation <1 g had a non-significant effect on the above results. Their meta-analysis included 17 studies, and the number of subjects included in each study ranged from 72 to 25,869. It is commendable that they included as many studies as possible to ensure the reliability of results. However, excessive studies with a small-sample size may lead to unreliable conclusions. It may explain why their results conflict with ours. Compared with the previous meta-analysis contains more RCTs, our research includes 14 RCTs. We strictly adhere to the inclusion criteria that the sample size of included RCTs must be >1,000. Our results were consistent in calculations by two effect models and Peter's test.

Two previous published meta-analyses provided information about the use of omega-3 FA supplementation in the secondary prevention of CHD (38, 39). However, they did not specify the past medical history and CHD development stages of included populations and included some RCTs with <100 participants. Our research confirmed previous conclusions and provided extra findings with four specific subgroup analyses. Our approach circumvents the aforementioned problems that occur in previously published analyses. First, we excluded RCTs with a small sample size and adopted two effect models to show the robustness of our results. Second, we conducted subgroup analysis of the included population to analyze the differences of omega-3 FA supplementation in cardiovascular events for primary or secondary prevention of CHD according to different stages of CHD development. Third, we performed subgroup analysis based on the usage dose of omega-3 FA supplementation, diabetes or not, and sex. Our research provided a better answer to the two questions of omega-3 FA supplementation that should be considered at which the development stage of CHD to prevent cardiovascular events and determine the proper dose of omega-3 FA supplementation.

This study encounters some limitations. (1) Although this study strictly followed the inclusion criteria and included experiments with more than 1,000 subjects, the number of studies included in some subgroup analyses was relatively small. More research is still required to support our results. (2) The RCTs included in this study are mostly performed by Western countries and lack sufficient data on Asians. (3) Subjects with CHD and acute MI may receive basic secondary prevention strategies, and whether secondary prevention measures would affect the clinical benefits of omega-3 FA supplementation cannot be excluded. The previously mentioned limitations require more large-scale RCTs to investigate further.

## Conclusion

This study conducted a meta-analysis of 14 large-scale RCTs to investigate the risk of cardiovascular events after receiving omega-3 FA supplementation. We found that omega-3 FA supplementation can reduce the risk of MACE, cardiovascular death, and MI. Additionally, it exhibits good clinical benefits for primary prevention and secondary prevention of CHD. Omega-3 FA supplementation application dose that ranges from 0.8 to 1.2 g exhibits more superiority than other doses in reducing cardiovascular risks.

## Data Availability Statement

The original contributions presented in the study are included in the article/Supplementary Material, further inquiries can be directed to the corresponding author.

## Author Contributions

SS and CG conceptualized the study, performed screening, data extraction, and data analysis by R software. Risk of bias was assessed by LZ and YX. Original draft preparation, reviewing, and editing were performed by SS, CG, and KJ. The work was supervised and funded by LM. All authors contributed to the article, approved, read, and agreed to the submitted version of the manuscript.

## Funding

This research was funded by National Natural Science Foundation of China, Grant Nos. 81870192 and 82170263.

## Conflict of Interest

The authors declare that the research was conducted in the absence of any commercial or financial relationships that could be construed as a potential conflict of interest.

## Publisher's Note

All claims expressed in this article are solely those of the authors and do not necessarily represent those of their affiliated organizations, or those of the publisher, the editors and the reviewers. Any product that may be evaluated in this article, or claim that may be made by its manufacturer, is not guaranteed or endorsed by the publisher.

## Supplementary Material

Supplementary Figure S1 Comparison of omega-3 FA supplementation vs. control group on the risks of stroke and revascularization.

Supplementary Table S1 PRISMA checklist.
