# Supplementary figures and images for: Omega-3 Fatty Acid Supplementation and Coronary Heart Disease Risks: A Meta-Analysis of Randomized Controlled Clinical Trials
Source: Front Nutr. 2022 Feb 3;9:809311. doi: 10.3389/fnut.2022.809311 (PMC8850984; doi:10.3389/fnut.2022.809311)

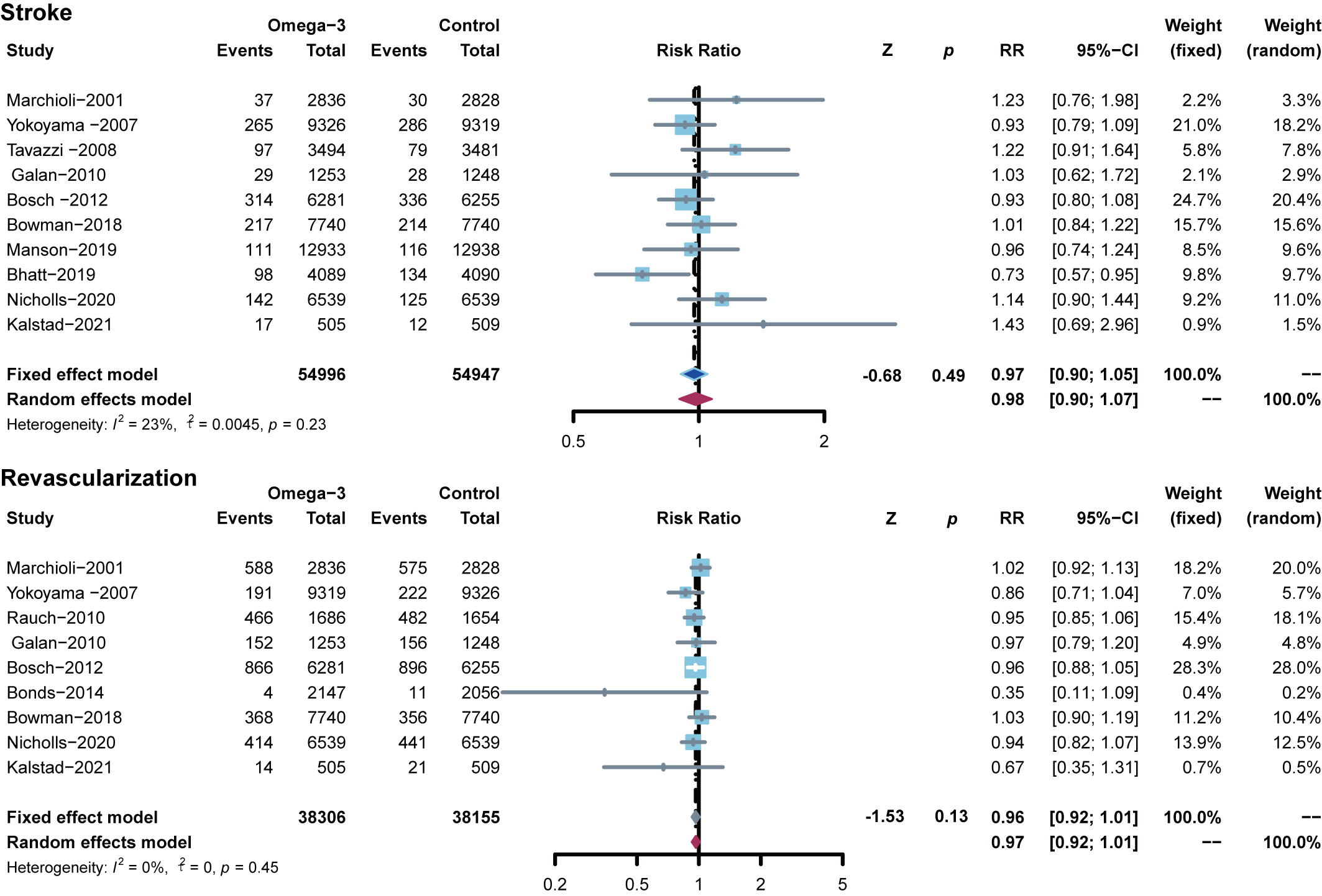

Supplement: Supplementary Figure S1 — Comparison of omega-3 FA supplementation vs. control group on the risks of stroke and revascularization. [file Image_1.TIF]
